# Real-time geometry-aware augmented reality in minimally invasive surgery

**DOI:** 10.1049/htl.2017.0068

**Published:** 2017-10-27

**Authors:** Long Chen, Wen Tang, Nigel W. John

**Affiliations:** 1 Department of Creative Technology, Bournemouth University, Poole, UK; 2 Deaprtment of Computer Science, University of Chester, Chester, UK

**Keywords:** endoscopes, biomedical optical imaging, augmented reality, medical image processing, image reconstruction, tumours, blood vessels, stereo image processing, real-time systems, surgery, real-time geometry-aware augmented reality, minimally invasive surgery, endoscopic surgery, reprojection error minimisation, three-dimensional mesh, zero mean normalised cross-correlation stereo-matching method, surface reconstruction, tumours, vessels

## Abstract

The potential of augmented reality (AR) technology to assist minimally invasive surgery (MIS) lies in its computational performance and accuracy in dealing with challenging MIS scenes. Even with the latest hardware and software technologies, achieving both real-time and accurate augmented information overlay in MIS is still a formidable task. In this Letter, the authors present a novel real-time AR framework for MIS that achieves interactive geometric aware AR in endoscopic surgery with stereo views. The authors’ framework tracks the movement of the endoscopic camera and simultaneously reconstructs a dense geometric mesh of the MIS scene. The movement of the camera is predicted by minimising the re-projection error to achieve a fast tracking performance, while the three-dimensional mesh is incrementally built by a dense zero mean normalised cross-correlation stereo-matching method to improve the accuracy of the surface reconstruction. The proposed system does not require any prior template or pre-operative scan and can infer the geometric information intra-operatively in real time. With the geometric information available, the proposed AR framework is able to interactively add annotations, localisation of tumours and vessels, and measurement labelling with greater precision and accuracy compared with the state-of-the-art approaches.

## 1 Introduction

Laparoscopic surgery is a minimally invasive surgery (MIS) procedure using endoscopes with small incisions to carry out internal operations on patients. While MIS offers considerable advantages over open surgeries, it also imposes big challenges on surgeons’ performance due to the well-known MIS issues associated with the field of view (FOV), hand–eye dis-alignment and disorientation. Augmented reality (AR) technology can help overcome the limitations by overlaying additional information with the real scene through augmentation of target surgical locations, annotations [1], labels [2], tumour measurement [3] or even three-dimensional (3D) reconstruction of anatomic structures [4, 5].

Despite recent advances in powerful miniaturised AR hardware devices and improvements on vision-based software algorithms, many issues in medical AR remain unsolved. Especially, the dramatic changes in tissue surface illumination and tissue deformation as well as the rapid movements of the endoscope during the insertion and the extrusion all give rise to a set of unique challenges that call for innovative approaches.

As with in any technological assisted medical procedures, accuracy of AR in MIS is paramount. With the use of traditional 2D feature based tracking algorithms such as those used in [1, 6–8], the rapid endoscope movement can easily cause feature points extracted from the vision algorithms to fall out of the FOV, resulting in poor quality visual guidance. The latest visual SLAM (simultaneous location and mapping) based approaches have the potential to overcome this issue by building an entire 3D map of the internal cavity of the MIS environment, but SLAM algorithms are often not robust enough when dealing with tissue deformations and scene illuminations [9–12]. Furthermore, in order to meet the demand of high computational performance, sparse landmark points are often used in MIS AR, and augmented information are mapped using planar detection algorithms such as random sample consensus (RANSAC) [13, 14]. As a result, AR content is mapped onto planes rather than curved organ surfaces.

In this Letter, we introduce a novel MIS AR framework that allows accurate overlay of augmented information onto curved surfaces, i.e. accurate annotation and labelling, and tumour measurements along the curved soft tissue surfaces. There are two key features of our proposed framework: (i) real-time computation of robust 3D tracking through a robust feature-based SLAM approach; (ii) an interactive geometry-aware AR environment through incrementally building a geometric mesh via zero mean normalised cross-correlation (ZNCC) stereo matching.

## 2 Related work

The traditional MIS AR approaches usually employ feature points tracking methods for information overlay. Feature-based 2D tracking methods such as Kanade–Lucas–Tomasi features [6, 7], scale-invariant feature transform (SIFT) [1], speeded up robust features (SURF) [15], even those methods specifically designed to cater for the scale, rotation and brightness of soft tissue [8] have a major drawback for AR, because selected feature points extracted from vision algorithms must be within the FOV. Therefore, traditional feature tracking methods can severely affect the precision of procedure guidance, especially in surgical scenes where the accuracy is paramount.

Recently, SLAM algorithms have led to new approaches to endoscopic camera tracking in MIS. Originally designed for robot navigation in unknown environments, the algorithms can be adapted for tracking the pose of endoscopic cameras while simultaneously building landmark maps inside the patient body during MIS procedures. SLAM-enabled AR systems not only improve the usability of AR in MIS due to no optical or magnetic tracking devices to obstruct the surgeons’ view, but they also offer greater accuracy and robustness compared with traditional feature-based AR systems.

Direct-based SLAM algorithms compare pixels [16] or reconstructed models [9, 17] of two images to estimate camera poses and reconstruct a dense 3D map by minimising the photometric errors. However, direct methods are more likely to fail when dealing with deformable scenes or when the illumination of the scene is inconsistent. Feature-based SLAM systems [10, 11] only compare a set of sparse feature points that are extracted from images. These methods estimate camera poses by minimising the re-projection error of the feature points. Therefore, feature-based SLAM methods are more suitable for MIS scenes due to its tolerance to illumination changes and small deformations. Feature-based SLAM such as extended Kalman filter (EKF)-SLAM has been used with laparoscopic image sequences [14, 18, 19] and a further motion compensation model [20] and stereo semi-dense reconstruction method [21] were integrated into the EKF-SLAM framework to deal with periodic deformation. However, the accuracy of EKF-SLAM tracking is not guaranteed and prone to inconsistent estimation and drifting due to the linearisation of motion and sensor models approximated by a first-order Taylor series expansion.

The first keyframe-based SLAM – PTAM (parallel tracking and mapping) [10] was a breakthrough in visual SLAM and has been used in MIS for stereoscope tracking [13]. The extension of PTAM – ORBSLAM [11] has also been tested on endoscope videos with map point densifying modifications [12], but the loss of accuracy still exists. Furthermore, since feature-based SLAM systems can only reconstruct maps based on sparse landmark points that barely describe the detailed 3D structure of the environment, the augmented AR content has to be mapped onto a plan through planar detection algorithms such as RANSAC [13]. Although feature-based SLAM is computationally efficient, different to real-life environments, in MIS scenes, flat surfaces are rare and organs and tissues do have smooth and curved surfaces, hence, resulting in inaccurate AR content registration. One example is the inaccurate labelling and measurement of tumour size without accurate surface fit for information overlay, which can be dangerous and misleading during MIS.

In this Letter, we present a novel real-time AR framework that provides 3D geometric information for accurate AR content registration and overlay in MIS. We propose a new approach to achieve robust 3D tracking through a feature-based SLAM for real-time performance and accuracy required for endoscopy camera tracking. To obtain accurate geometric information, we incrementally build a dense 3D point cloud by using ZNCC stereo matching. Therefore, our framework handles the challenging situations of rapid endoscopy movements with robust real-time tracking, while providing an interactive geometry-aware AR environment.

## 3 Methods

**Fig. 1:**
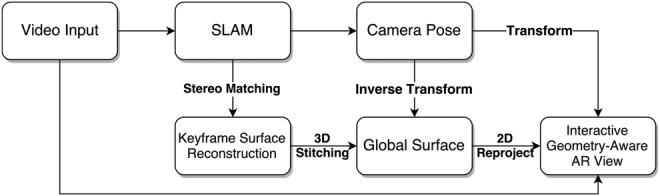
Flowchart describing the whole framework

As can be seen from the flowchart in Fig. 1, our proposed framework starts with a SLAM system that can track and estimate the camera pose frame by frame. The following stereo-matching algorithm based on ZNCC is used to reconstruct dense surface at each keyframe, which is then transformed and stitched to a global surface based on the inverse transformation of the camera pose. Finally, the global surface is re-projected to 2D based on the camera pose and overlaid on the image frame, serving as an interactive geometric layer. The geometric layer enables the interactive AR applications such as online measurement which will be explained in Section 4.

### 3.1 Landmark point detection and triangulation

In medical interventions, real-time performance and accuracy are both critical. We adopt oriented FAST and rotated BRIEF (ORB) [22] feature descriptors for feature points extraction, encoding and comparison to match landmark points in left and right stereo images. ORB is a binary feature point descriptor that is an order of magnitude faster than SURF [23], more than two orders faster than SIFT [24] and also offers better accuracy than SURF and SIFT [22]. In addition, ORB features are invariant to rotation, illumination and scale, hence, capable of dealing with challenge endoscope camera scenes (rapid rotating, zooming and changing of brightness).

**Fig. 2:**
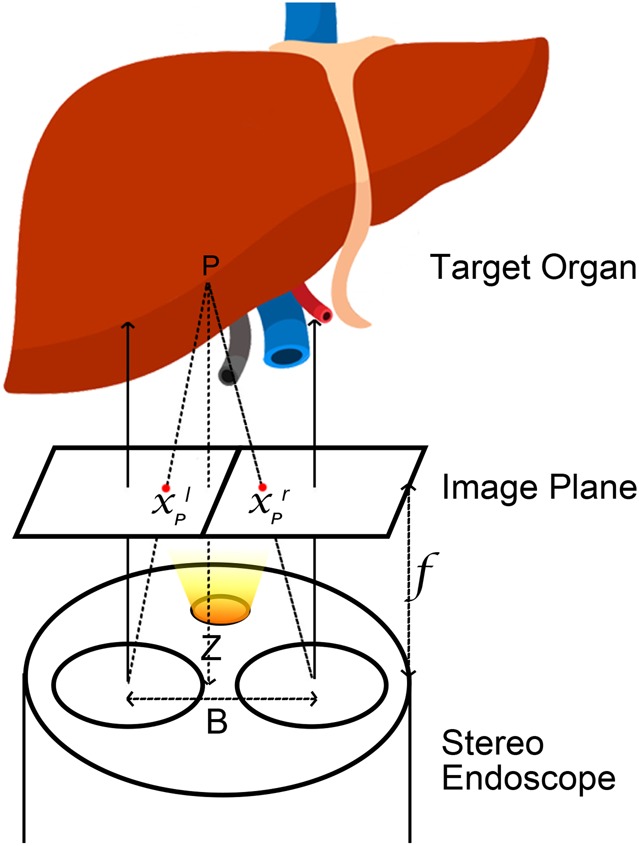
By using a stereo endoscope, the 3D position of any point in the view can be directly estimated by using stereo triangulation

We apply the ORB detector and find the matched keypoints on left and right images. Let $x_P^l $ and $x_P^r $ be the *x* coordinates on the left and right images, respectively. Assuming the left image and the right image are already rectified, the focal length of both cameras *f* and the baseline *B* are known fixed values, by similar triangles, the depth or the perpendicular distance *Z* between the points and the endoscope can be found according to similar triangles (see Fig. 2)

(1) $$\displaystyle{{B - \left({x_P^l - x_P^r } \right)} \over {Z - f}} = \displaystyle{B \over Z}\quad \Rightarrow \quad Z = \displaystyle{{\,f \cdot B} \over {d_P}}\eqno\lpar 1\rpar $$

where $x_P^l - x_P^r $ is the disparity $d_P$ of the two corresponding keypoints in the left and the right images detected by the ORB feature.

We then perform a specular reflection detection by removing the keypoints that have intensities above a threshold for efficiency. This could effectively remove the influence of specular reflections from the next stage of computation.

### 3.2 Frame-by-frame camera pose estimation

Any AR application requires the real-time frame-by-frame tracking to continuously update the overlay positions. To meet the real-time requirement, after initialisation, we employ the constant velocity motion model used by MonoSLAM [25] to roughly estimate the position $r_{t + 1}$ and quaternion rotation $q_{t + 1}$ of the camera position based on the current linear velocity $v_t$ and angular velocity $w_t$ in a small period ${\rm \Delta }t$

(2) $$\left. {\matrix{ {r_{t + 1} = r_t + v_t \cdot {\rm \Delta }t} \cr {q_{t + 1} = q_t \times q\left({\omega _t \cdot {\rm \Delta }t} \right)} \cr {v_t = v_{t - 1} + a_{t - 1} \cdot {\rm \Delta }t} \cr {\omega _t = \omega _{t - 1} + \alpha _{t - 1} \cdot {\rm \Delta }t} \cr } } \right\}\eqno\lpar 2\rpar $$

Based on the predicted camera pose $\left({r_{t + 1}\comma \; q_{t + 1}} \right)$, the potential regions where the feature points may appear on the image are estimate by re-projection of 3D points, hence reducing searching areas and computational cost.

A RANSAC procedure is then performed to obtain the rotation and translation estimations from the set of all the inlier points. During each RANSAC iteration, three pairs of corresponding 3D points from current point set $p_t^i $ and point set in next period $p_{t + 1}^i $ are selected randomly to calculate the rotation matrix ${\bi R}$ and the translation ***T***, which minimises the following objective function:

(3) $$\min \mathop \sum \limits_{i = 1}^n \left\Vert {\,p_t^i - \left({{\bi R} \times p_{t + 1}^i + {\bi T}} \right)} \right\Vert \eqno\lpar 3\rpar $$

From the set with smallest re-projection error, the set of outlier points is rejected and all the inliers are used for a refinement of the final rotation and translation estimations.

During the inlier/outlier identification scheme by RANSAC, false matched ORB feature points, moving specular reflection points and deforming points are effectively rejected. This is a very important step for a MIS scene where the tissue deformation caused by respiration and heartbeat, as well as blood, smoke and surgical instruments can have impact on the tracking stability. Therefore, at this stage, we use the strategy to filter out any influence caused by occlusion and deformation to recover the camera pose. Indeed, the deformable surface is an unsolved challenge in MIS AR; we address this issue by reconstructing a dense 3D map through a more efficient stereo-matching method (see Section 3.4).

### 3.3 Keyframe-based bundle adjustment (BA)

As our camera pose estimation is only based on the last state, the accumulation of error over time would cause system drifting. However, we cannot perform a global optimisation for every frame as this will slow down the system over time. We follow the successful approach of PTAM [10] and ORBSLAM [11] in correcting system drafting, which use the keyframe-based SLAM framework to save ‘snapshots’ of some frames as keyframes to enhance the robustness of the tracking whilst not increasing computational load on the system. Each keyframe is selected based on the criteria that the common keypoints of the two keyframes are <80% keypoints but the total number exceeds 50.

Once a keyframe is assigned, BA is applied to refine the 3D positions of each stored keyframe ${\rm K}{\rm F}_i$ and the landmark points $P_j$ by minimising the total Huber robust cost function w.r.t. the re-projection error between 2D matched keypoints $p_{i\comma j}$ and camera perspective projections of the 3D positions of keyframes ${\rm K}{\rm F}_i$ and the landmark points $P_j$

(4) $$\mathop {\arg {\rm \; }\min }\limits_{{\rm K}{\rm F}_i\comma P_j} {\rm \; }\mathop \sum \limits_{i\comma j} \rho _h\left({\left\Vert {\,p_{i\comma j} - {\rm CamProj}\left({{\rm K}{\rm F}_i\comma \; P_j} \right)} \right\Vert } \right)\eqno\lpar 4\rpar $$

### 3.4 ZNCC dense stereo matching

We create a feature-based visual odometry system for the endoscopic camera tracking and landmark points mapping, which takes into account of illumination changes, specular reflections and tissue deformations in MIS scenes. However, as the sparse landmark points can barely describe the challenging environment of MIS scenes, we perform a dense stereo matching upon the landmark points to create a dense reconstruction result.

The dissimilarity measure used during the stereo matching is a patch-based ZNCC method. The cost value $C\left({\,p\comma \; d} \right)$ for a pixel *p* at disparity *d* is derived by measuring the ZNCC of the pixel in the left image and the corresponding pixel $p - d$ in the right image

(5) $$ {C\left({p\comma \; d} \right)= \displaystyle{{\mathop \sum \nolimits_{q \in N_p} \left({I_L\left(q \right)- {\bar I}_L\left(\,p \right)} \right)\cdot \left({I_R\left({q - d} \right)- {\bar I}_R\left({\,p - d} \right)} \right)} \over {\sqrt {\mathop \sum \nolimits_{q \in N_p} {\left({I_L\left(q \right)- {\bar I}_L\left(\,p \right)} \right)}^2 \cdot \mathop \sum \nolimits_{q \in N_p} {\left({I_R\left({q - d} \right)- {\bar I}_R\left({\,p - d} \right)} \right)}^2} }}}\eqno\lpar 5\rpar $$

where $\bar I\left(\,p \right)= \lpar 1/N_p\rpar \sum\nolimits_{q \in N_p} {I\left(q \right)} $ is the mean intensity of the patch $N_p$ centred at *p*.

ZNCC is proven to be less sensitive to illumination changes and can be parallelised efficiently on a graphics processing unit (GPU) [26]. A winner-takes-all strategy is applied to choose the best disparity value for each pixel *p*, followed by a convex optimisation to solve the cost volume constructed by Huber-$L^1$ variational energy function [27] for a smooth disparity map. We used the GPU implement of ZNCC and convex optimisation for the efficient disparity map estimation and filtering in real time.

### 3.5 Incremental building of geometric mesh

**Fig. 3:**
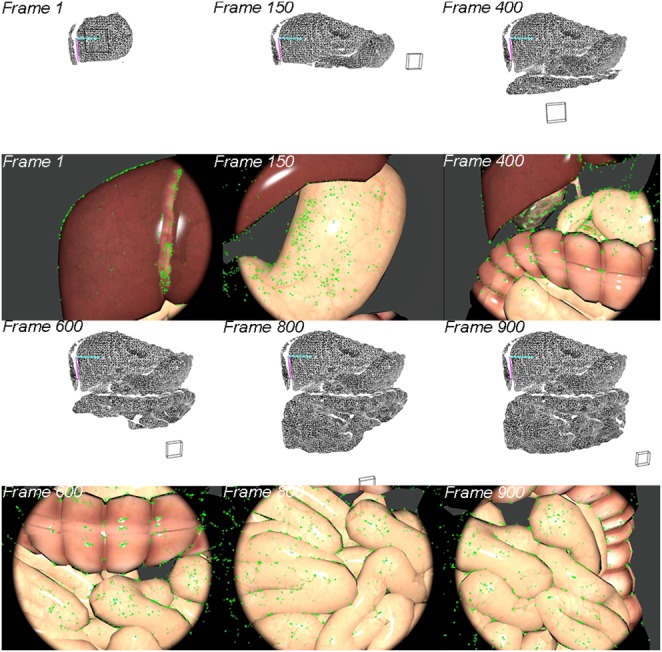
Incrementally building the geometric mesh. Rectangular boxes are the estimated camera pose; green points are detected landmark points

**Fig. 4:**
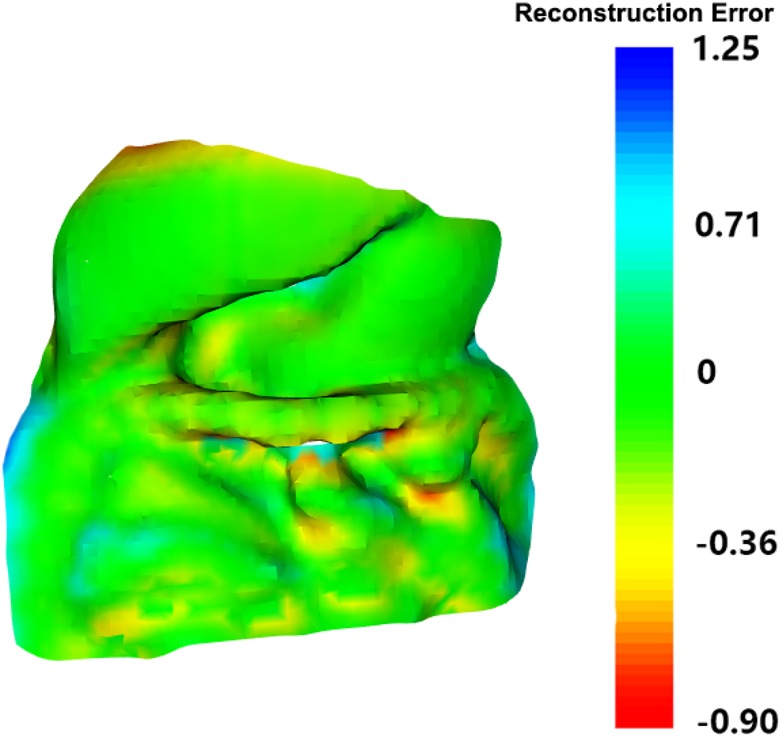
Reconstruction error map

**Fig. 5:**
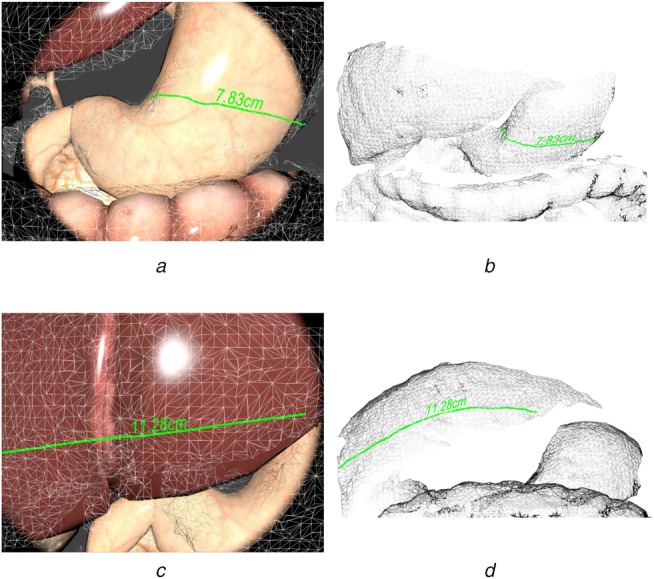
Measurement application of our proposed geometry-aware AR framework. Note that the measuring lines (green lines) accurately follow along the curve surface

The 3D dense points estimated by stereo matching are transformed to the world coordinate system by the transformation matrix from frame space to the world space $T_{\,f2w}$ that was estimated by our feature-based SLAM system. A fast triangulation method [28] is then used to incrementally reconstruct the dense points into a surface mesh. Fig. 3 demonstrates the incrementally building process from frames 1 to 900. The first and third rows are the reconstructed geometric mesh while the second and fourth rows are the current video frames. The geometric mesh can be built incrementally to form a global mesh that can then be re-projected back to the camera's view using the estimated camera pose for the augmented view (see Figs. 4*a*, *c* and 5*a*, *c*).

## 4 Results and discussion

We have designed a two-parts assessment process to evaluate our AR framework: (i) using a realistic 3D simulated MIS scene as the ground truth study to measure the reconstruction error by measuring the difference between the ground truth values and the reconstructed values; (ii) using a real in vivo video acquired from the Hamlyn Centre Laparoscopic/Endoscopic Video Datasets [29, 30] to assess the quality of applications of our proposed framework, i.e. measurements, adding AR labels and areas highlighting.

### 4.1 System setup

Our system is implemented in an Ubuntu 14.04 environment using C/C++. All experiments are conducted on a workstation equipped with Intel Xeon(R) 2.8 GHz quad core CPU, 32 GB memory and one NVIDIA GeForce GTX 970 graphics card. The size of the simulation image sequences and in vivo endoscope videos is 840 × 640 pixels. The AR framework and 3D surface reconstruction run in different threads. The 3D surface reconstruction process takes about 200 ms to traverse the entire pipeline for each frame. Our proposed AR framework can run in real time at 26 fps when the reconstruction only performs at keyframes.

### 4.2 Ground truth study using simulation data

The performance of our proposed framework is measured in terms of reconstruction accuracy by comparing the reconstructed surface with the 3D model used to render the simulation video.

To quantitatively evaluate the performance of the progressive reconstruction result, we used Blender [31] – an open source 3D software to render realistic image sequences of a simulated abdominal cavity scene using a set of pre-defined endoscopic camera movements. The simulated scene contains models scaled to real-life size according to an average measured liver diameter of 14.0 cm [32], and the digestive system is rendered with appropriate textures to make the scene as realistic as possible. The material property is set with a strong specularity component to simulate the smooth and reflective liver surface tissue. The luminance is intentionally set high to simulate an endoscope camera as shown in Fig. 5 with a realistic endoscopic lighting condition by using a spot light attached to the main camera. We have designed a camera trajectory that hovers around the 3D models. There are a total of 900 frames of image sequences at a frame rate of 30 fps being rendered, which is equivalent to a 30 s video.

Root mean square distance (RMSD) is used to evaluate the overall distance between the simulated and the reconstructed surfaces. By aligning the surfaces to the real-world coordinate system, we apply a grid sample to get a series of *x*, *y* coordinate points based on the surface area, and then compared the distance of the *z-*value of the two surfaces

$${\rm RMSD} = \sqrt {\displaystyle{1 \over {mn}}\mathop \sum \limits_{x = 1}^m \mathop \sum \limits_{y = 1}^n {\left({Z_{x\comma y} - z_{x\comma y}} \right)}^2} $$

The RMSD measurement for the two surface alignments has shown a good surface reconstruction results from our proposed methods, compared with the ground truth surface, the RMSD is 2.37 mm. The reconstruction error map can be viewed in Fig. 4.

### 4.3 Real endoscopic video evaluation

**Fig. 6:**
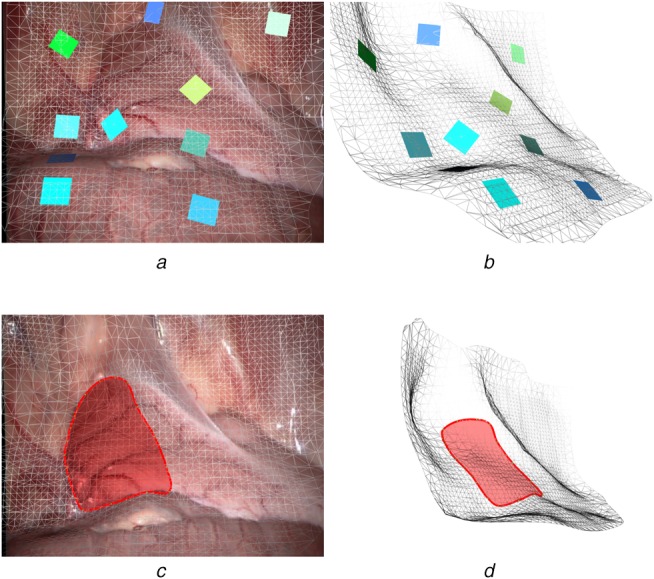
Applications of our proposed geometry-aware AR framework *a* Adding AR labels according to the norm of the geometric surface *b* Side view of labels in mesh view *c* Area highlight and measurement *d* Side view of highlighted area in mesh view

To qualitatively evaluate the performance of our proposed surface reconstruction framework, we applied the proposed approach on in vivo videos that we acquired from Hamlyn Centre Laparoscopic/Endoscopic Video Datasets [29, 30]. Fig. 6*a* shows the reconstruction result from our 3D reconstruction framework with the augmented view of in vivo video sequences. By clicking the mesh, augmented objects (coloured planes) can be superimposed at corresponding positions with correct poses based on the normals of the points at the click locations. Fig. 6*b* shows the side view of the mesh; note that the coloured planes (which could be labels) are sticking onto the mesh correctly to create a realistic augmented environment. Fig. 6*c* shows the area highlighting function of our proposed AR framework. Fig. 6*d* is the corresponding mesh view. The area highlighting function can be extended to an area measurement and line measurement (such as shown in Fig. 5) application once the extrinsic parameters of the camera are known.

## 5 Conclusions

In this Letter, we presented a novel AR framework for MIS. Our framework handles the two intertwined issues of tracking the rapid endoscope camera movements and providing accurate information overlay onto the curved surfaces of organs and tissues. By adapting the latest SLAM algorithms, we take a set of innovative approaches at the each stage of the AR process to improve the computational performances and AR registration accuracy. As a result, an interactive real-time geometric aware AR system has been developed. The system is capable of dealing with small soft tissue deformations, rapid endoscope movement and illumination change, which are common challenges in MIS AR. Our proposed system does not require any prior template or pre-operative scan. The system can overlay accurate augmented information such as annotations, labelling and measurements of a tumour over curved surfaces, greatly improving the quality of AR technology in MIS.

In future work, we will carry out a clinical pilot study. A case scenario will be investigated in collaboration with a practicing surgeon, and comparisons will be made as to the effectiveness of our system with the current procedural approach used.

## 6 Funding and declaration of interests

Conflict of interest: none declared.

## References

[1] Kim J.-H. Bartoli A. Collins T.: ‘Tracking by detection for interactive image augmentation in laparoscopy’. Lecture Notes in Computer Science, 2012, pp. 246–255

[2] Su L.-M. Vagvolgyi B.P. Agarwal R.: ‘Augmented reality during robot-assisted laparoscopic partial nephrectomy: toward real-time 3D-CT to stereoscopic video registration’, Urology, 2009, 73, (4), pp. 896–900 (doi: 10.1016/j.urology.2008.11.040) 19193404 10.1016/j.urology.2008.11.040

[3] Bourdel N. Collins T. Pizarro D.: ‘Use of augmented reality in laparoscopic gynecology to visualize myomas’, Fertility Sterility, 2017, 107, pp. 737–739 (doi: 10.1016/j.fertnstert.2016.12.016) 28089570 10.1016/j.fertnstert.2016.12.016

[4] Haouchine N. Dequidt J. Peterlik I.: ‘Image-guided simulation of heterogeneous tissue deformation for augmented reality during hepatic surgery’. IEEE Int. Symp. on Mixed and Augmented Reality (ISMAR), 2013, 2013, pp. 199–208

[5] Haouchine N. Cotin S. Peterlik I.: ‘Impact of soft tissue heterogeneity on augmented reality for liver surgery’, IEEE Trans. Vis. Comput. Graph., 2015, 21, (5), pp. 584–597 (doi: 10.1109/TVCG.2014.2377772) 26357206 10.1109/TVCG.2014.2377772

[6] Du X. Clancy N. Arya S.: ‘Robust surface tracking combining features, intensity and illumination compensation’, Int. J. Comput. Assist. Radiol. Surg., 2015, 10, (12), pp. 1915–1926 (doi: 10.1007/s11548-015-1243-9) 26100122 10.1007/s11548-015-1243-9

[7] Planteféve R. Peterlik I. Haouchine N.: ‘Patient-specific biomechanical modeling for guidance during minimally-invasive hepatic surgery’, Ann. Biomed. Eng., 2016, 44, (1), pp. 139–153 (doi: 10.1007/s10439-015-1419-z) 26297341 10.1007/s10439-015-1419-z

[8] Mountney P. Yang G.-Z.: ‘Soft tissue tracking for minimally invasive surgery: learning local deformation online’, Med. Image Comput. Comput. Assist. Interv., 2008, 11, (Pt 2), pp. 364–372 18982626 10.1007/978-3-540-85990-1_44

[9] Turan M. Almalioglu Y. Araujo H.: ‘A non-rigid map fusion-based RGB-depth SLAM method for endoscopic capsule robots’. ArXiv e-prints, May 2017 10.1007/s41315-017-0036-4 PMC5727175 29250588

[10] Klein G. Murray D.: ‘Parallel tracking and mapping for small AR workspaces’. Proc. 6th IEEE and ACM Int. Symp. Mixed and Augmented Reality, November 2007, pp. 225–234

[11] Mur-Artal R. Montiel J.M.M. Tardós J.D.: ‘Orb-SLAM: a versatile and accurate monocular SLAM system’, IEEE Trans. Robot., 2015, 31, (5), pp. 114–1163 (doi: 10.1109/TRO.2015.2463671)

[12] Hostettler A. Doignon C. Soler L.: ‘Orbslam-based endoscope tracking and 3d reconstruction’. MICCAI 2016 CARE, 2016

[13] Lin B. Johnson A. Qian X.: ‘Simultaneous tracking, 3D reconstruction and deforming point detection for stereoscope guided surgery’. Lecture Notes in Computer Science, 2013, pp. 35–44

[14] Grasa O.G. Bernal E. Casado S.: ‘Visual slam for handheld monocular endoscope’, IEEE Trans. Med. Imag., 2014, 33, (1), pp. 135–146 (doi: 10.1109/TMI.2013.2282997) 10.1109/TMI.2013.2282997 24107925

[15] Kumar A. Wang Y.-Y. Wu C.-J.: ‘Stereoscopic visualization of laparoscope image using depth information from 3D model’, Comput. Methods Programs Biomed., 2014, 113, (3), pp. 862–868 (doi: 10.1016/j.cmpb.2013.12.013) 24444752 10.1016/j.cmpb.2013.12.013

[16] Engel J. Schï£ips T. Cremers D.: ‘LSD-SLAM: large-scale direct monocular SLAM’. Computer Vision – ECCV 2014, 2014, pp. 834–849

[17] Chang P.-L. Handa A. Davison A.J.: ‘Robust real-time visual odometry for stereo endoscopy using dense quadrifocal tracking’. Int. Conf. on Information Processing in Computer-Assisted Interventions, 2014, pp. 11–20

[18] Mountney P. Stoyanov D. Davison A.: ‘Simultaneous stereoscope localization and soft-tissue mapping for minimal invasive surgery’. Medical Image Computing and Computer-Assisted Intervention – MICCAI 2006, January 2006 10.1007/11866565_43 17354909

[19] Mountney P. Yang G.Z.: ‘Dynamic view expansion for minimally invasive surgery using simultaneous localization and mapping’. 2009 Annual Int. Conf. of the IEEE Engineering in Medicine and Biology Society, September 2009, pp. 1184–1187 10.1109/IEMBS.2009.5333939 19964502

[20] Mountney P. Yang G.-Z.: ‘Motion compensated slam for image guided surgery’. Medical Image Computing and Computer-Assisted Intervention – MICCAI 2010, January 2010 10.1007/978-3-642-15745-5_61 20879352

[21] Totz J. Mountney P. Stoyanov D.: ‘Dense surface reconstruction for enhanced navigation in MIS’. Medical Image Computing and Computer-Assisted Intervention – MICCAI 2011, January 2011 10.1007/978-3-642-23623-5_12 22003604

[22] Rublee E. Rabaud V. Konolige K.: ‘Orb: an efficient alternative to sift or surf’. IEEE Int. Conf. on Computer Vision (ICCV), 2011, 2011, pp. 2564–2571

[23] Bay H. Tuytelaars T. Van Gool L.: ‘Surf: speeded up robust features’. Computer Vision – ECCV 2006, 2006, pp. 404–417

[24] Lowe D.G.: ‘Distinctive image features from scale-invariant keypoints’, Int. J. Comput. Vis., 2004, 60, (2), pp. 91–110 (doi: 10.1023/B:VISI.0000029664.99615.94)

[25] Davison A.J. Reid I.D. Molton N.D.: ‘Monoslam: real-time single camera SLAM’, IEEE Trans. Pattern Anal. Mach. Intell., 2007, 29, (6), pp. 1052–1067 (doi: 10.1109/TPAMI.2007.1049) 17431302 10.1109/TPAMI.2007.1049

[26] Stoyanov D. Scarzanella M.V. Pratt P.: ‘Real-time stereo reconstruction in robotically assisted minimally invasive surgery’. Medical Image Computing and Computer-Assisted Intervention – MICCAI 2010, January 2010 10.1007/978-3-642-15705-9_34 20879241

[27] Chang P.-L. Stoyanov D. Davison A.J.: ‘Real-time dense stereo reconstruction using convex optimisation with a cost-volume for image-guided robotic surgery’. Medical Image Computing and Computer-Assisted Intervention – MICCAI 2013, January 2013 10.1007/978-3-642-40811-3_6 24505647

[28] Marton Z.C. Rusu R.B. Beetz M.: ‘On fast surface reconstruction methods for large and noisy point clouds’. 2009 IEEE Int. Conf. on Robotics and Automation, May 2009, pp. 3218–3223

[29] Imperial College London: ‘Hamlyn centre laparoscopic/endoscopic video datasets’, 2016 Available at http://hamlyn.doc.ic.ac.uk/vision/, accessed 6 November 2016

[30] Mountney P. Stoyanov D. Yang G.-Z.: ‘Three-dimensional tissue deformation recovery and tracking’, IEEE Signal Process. Mag., 2010, 27, (4), pp. 14–24 (doi: 10.1109/MSP.2010.936728)

[31] Blender: ‘Blender – free and open 3d creation software’, 2016, Available at https://www.blender.org/, accessed 6 November 2016

[32] Kratzer W. Fritz V. Mason R.A.: ‘Factors affecting liver size: a sonographic survey of 2080 subjects’, J. Ultrasound Med., Off. J. Am. Inst. Ultrasound Med., 2003, 22, pp. 1155–1161 (doi: 10.7863/jum.2003.22.11.1155) 10.7863/jum.2003.22.11.1155 14620885

